# Evaluating MAPT p.A152T as a risk factor for the 3R tauopathy Pick’s disease

**DOI:** 10.1093/braincomms/fcag266

**Published:** 2026-07-09

**Authors:** Nicole Tamvaka, William Scotton, Meredith T Lilley, Maryam Shoai, Marios Gavrielatos, Michael G Heckman, Alexandra I Soto-Beasley, Shanu F Roemer, Matthew C Baker, Rosa Rademakers, Melissa E Murray, Dennis W Dickson, John A Hardy, Casey N Cook, Jonathan D Rohrer, Owen A Ross, Pick’s disease international consortium, Rebecca R Valentino, Tammaryn Lashley, Hannah L Macpherson, Thomas T Warner, Zane Jaunmuktane, Alejandro Martinez-Carrasco, Kin Mok, Tamas Revesz, Elizabeth A Christopher, Michael DeTure, Shunsuke Koga, Neill R Graff-Radford, Bradley F Boeve, Keith A Josephs, Ronald C Petersen, Jennifer Whitwell, Ranjan Duara, Kelly E Lyons, Rajesh Pahwa, Lea T Grinberg, William W Seeley, Bruce Miller, Athena Schlereth, Salvatore Spina, David J Irwin, David A Wolk, EunRan Suh, Vivianna M Van Deerlin, Edward B Lee, Matthew P Frosch, Theresa R Connors, Derek H Oakley, Laura Molina-Porcel, Iban Aldecoa, Mircea Balasa, Sergi Borrego-Ecija, Raquel Sanchez-Valle, Jordi Gascon-Bayarri, Pilar Sanz-Cartagena, Gerard Pinol-Ripoll, Rosa Maria de Eugenio Huélamo, Ellen Gelpi, Tamar Gefen, Rudolph J Castellani, Margaret E Flanagan, Emily J Rogalski, Sandra Weintraub, Julie A Schneider, Kevin F Bieniek, Xiongwei Zhu, Javier Redding-Ochoa, Koping Chang, Juan C Troncoso, Stefan Prokop, Bernardino Ghetti, Kathy L Newell, Max Jacobsen, Andrew C Robinson, Federico Roncaroli, Christopher Kobylecki, Matthew Jones, Anna Richardson, Julie Snowden, James B Rowe, Annelies Quaegebeur, Kieren Allinson, Thomas G Beach, Geidy E Serrano, Andrew F Teich, Xena E Flowers, Allison C Heaps, Sandra P Leskinen, Julia L Keith, Sandra E Black, Mario Masellis, Andrew King, Safa-Al Sarraj, Claire Troakes, Glenda M Halliday, John R Hodges, Jillian J Kril, John B Kwok, Olivier Piguet, Marla Gearing, Sigrun Roeber, Christopher M Morris, Johannes Attems, Alan J Thomas, Charles L White, Bret M Evers, Naguib Mechawar, Susana Boluda, Danielle Seilhean, Isabelle Le Ber, Sabrina Turbant-Leclere, Ian R MacKenzie, Anne A Sieben, Patrick P Cras, Bart B De Vil, P P De Deyn, C Dirk Keene, Caitlin S Latimer, Aimee Schantz, Thomas D Bird, Vahram Haroutunian, Dushyant Purohit, Jamie M Walker, Robert Rissman, Catriona McLean, Matthew D Cykowski, Shih-Hsiu J Wang, John F Ervin, Caroline Graff, Inger Nennesmo, Rashed M Nagra, James Riehl, Gabor G Kovacs, Giorgio Giaccone, Manuela Neumann, Lee-Cyn Ang, Elizabeth C Finger, Agostinho Carvalho, Cristina Cunha, Huw R Morris, Cornelis Blauwendraat, Mike A Nalls, Andrew B Singleton, Dan Vitale, Richard J Perrin, Anne M Chaffee, Zbigniew K Wszolek

**Affiliations:** Department of Neuroscience, Mayo Clinic, Jacksonville, FL 32224, USA; Dementia Research Centre, Department of Neurodegenerative Disease, University College London, Queen Square Institute of Neurology, London WC1E 6BT, UK; Department of Neuroscience, Mayo Clinic, Jacksonville, FL 32224, USA; Neuroscience Graduate Program, Mayo Clinic Graduate School of Biomedical Sciences, Jacksonville, FL 32224, USA; Department of Neurodegenerative Disease, University College London, Queen Square Institute of Neurology, London WC1N 3BG, UK; Department of Neuroscience, Mayo Clinic, Jacksonville, FL 32224, USA; Division of Clinical Trials and Biostatistics, Mayo Clinic, Jacksonville, FL 32224, USA; Department of Neuroscience, Mayo Clinic, Jacksonville, FL 32224, USA; Department of Neuroscience, Mayo Clinic, Jacksonville, FL 32224, USA; Department of Neuroscience, Mayo Clinic, Jacksonville, FL 32224, USA; Department of Neuroscience, Mayo Clinic, Jacksonville, FL 32224, USA; Department of Biomedical Sciences, University of Antwerp, Antwerp 2610, Belgium; VIB Center for Molecular Neurology, VIB, Antwerp 2610, Belgium; Department of Neuroscience, Mayo Clinic, Jacksonville, FL 32224, USA; Department of Neuroscience, Mayo Clinic, Jacksonville, FL 32224, USA; Department of Neurodegenerative Disease, University College London, Queen Square Institute of Neurology, London WC1N 3BG, UK; Reta Lila Weston Institute, University College London, Queen Square Institute of Neurology, London WC1N 1PJ, UK; UK Dementia Research Institute at UCL, London NW1 3BT, UK; Institute for Advanced Study, The Hong Kong University of Science and Technology, Hong Kong, China; Department of Neuroscience, Mayo Clinic, Jacksonville, FL 32224, USA; Neuroscience Graduate Program, Mayo Clinic Graduate School of Biomedical Sciences, Jacksonville, FL 32224, USA; Department of Molecular Medicine, USF Health, Morsani College of Medicine, Tampa, FL 33602, USA; Dementia Research Centre, Department of Neurodegenerative Disease, University College London, Queen Square Institute of Neurology, London WC1E 6BT, UK; Department of Neuroscience, Mayo Clinic, Jacksonville, FL 32224, USA; Neuroscience Graduate Program, Mayo Clinic Graduate School of Biomedical Sciences, Jacksonville, FL 32224, USA; Department of Clinical Genomics, Mayo Clinic, Jacksonville, FL 32224, USA

**Keywords:** Pick’s disease, tauopathy, MAPT p.A152T, rare variant

## Abstract

Genetic studies have significantly advanced our understanding of tauopathies, yet the genetic aetiology of Pick’s disease, a rare 3-Repeat tauopathy, remains unclear. The MAPT p.A152T variant has been identified as a risk factor for Alzheimer’s disease and progressive supranuclear palsy, but its role in Pick’s disease is unknown. In this study, we examined the prevalence of MAPT p.A152T in the largest series of neuropathologically confirmed Pick’s disease cases to date (*n* = 401). Through genotyping, we identified a single mutation carrier in the Pick’s disease cohort (minor allele frequency = 0.12%). We previously reported MAPT p.A152T at a 0.20% frequency in healthy controls (*n* = 2456), suggesting that it does not associate with 3-Repeat tauopathy risk. To further investigate the effect of the variant on *MAPT* transcript expression, we used bulk RNA sequencing in Alzheimer’s disease and progressive supranuclear palsy A152T mutation carriers. We did not detect significant differences in 4-Repeat tau levels, though preliminary trends may indicate more nuanced effects that need to be examined with long-read sequencing in a larger series. Overall, our study suggests that MAPT p.A152T does not increase Pick’s disease risk and may instead be linked to 4-Repeat or mixed tau pathologies, warranting further functional investigation.

## Teaser

Tamvaka *et al*. report that the MAPT p.A152T variant is rare in the largest series of pathologically confirmed Pick’s disease cases and is not associated with disease risk. Their findings suggest that A152T may instead relate to other tau pathologies, warranting further functional investigation.

## Graphical Abstract

**Graphical Abstract:**
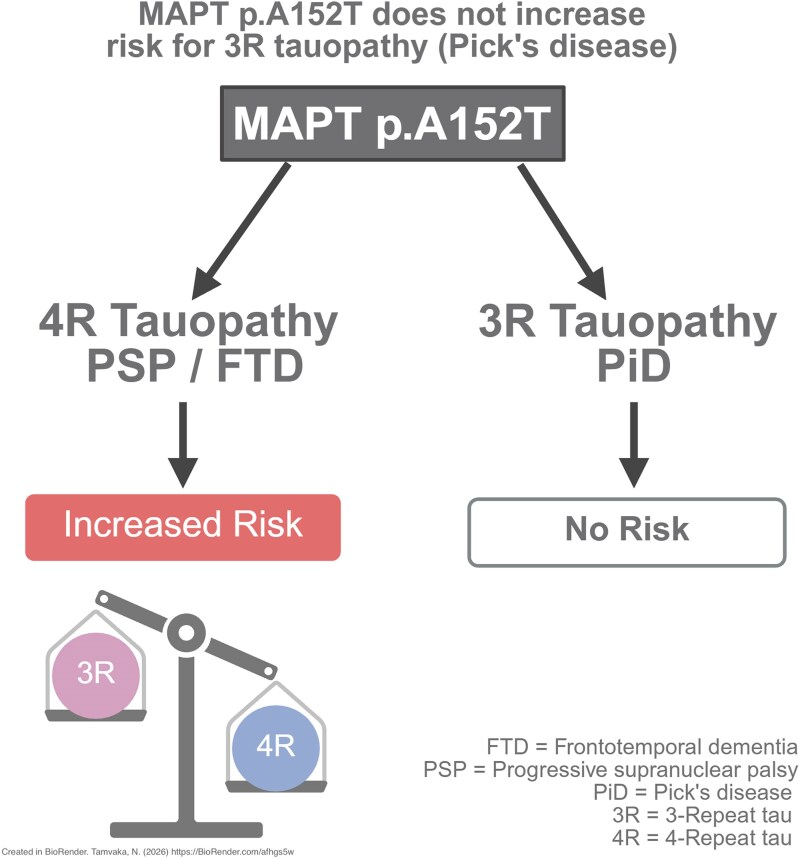

## Introduction

Understanding variation at the *MAPT* locus has been of great importance to the study of neurodegeneration as it strongly contributes to the pathogenesis of a myriad of disorders. Common variation defining the *MAPT* H1 extended haplotype is considered to be a major risk factor for frontotemporal lobar degeneration spectrum disorders (FTLD), progressive supranuclear palsy (PSP) and corticobasal degeneration (CBD).^1-3^ At the same time, the H1 haplotype and the common alleles that define it have been linked to an increased risk of other neurodegenerative diseases, including Parkinson’s disease (PD) and Alzheimer’s disease (AD).^4,5^ In addition, the less common H2 haplotype was recently associated with increased risk of Pick’s disease (PiD), another FTLD disorder with tau pathology.^6^

In addition to common variation, rare *MAPT* mutations have also been identified in families with FTLD with tau pathology (FTLD-tau).^7,8^ Since the discovery of the first *MAPT* missense and splice variants,^7^ there have been over 100 mutations identified that are predicted to have different levels of pathogenicity across the tauopathies.^9,10^ Some of the identified variants are highly penetrant, disease-causing mutations that account for 5–20% of FTLD-tau.^11^ Other variants have been identified in individuals with sporadic disease and have been shown to have intermediate effect size and act as risk factors or disease modifiers. One such example is the rare p.A152T variant located in exon 7 of the *MAPT* gene known to increase risk for both AD and FTLD as well as atypical tauopathies.^12-14^ As such, it is evident that *MAPT* harbours both Mendelian pathogenic variation and rare and common variants acting as disease risk factors.

*MAPT* variants are linked to various phenotypic and neuropathological entities and are associated with distinct disease processes, including microtubule binding and assembly, alternative splicing and aggregation propensity.^9^ Moreover, variants have also been evaluated for their effect on tau isoform-specific aggregation since the tau protein exists in many different isoforms in the adult human brain. These isoforms are classified into 3-Repeat (3R) and 4-Repeat (4R) forms based on the number of repeat motifs present in the microtubule-binding domain of the protein. This domain is encoded by four highly similar exons (9, 10, 11 and 12) with the splicing of exon 10 at the mRNA level differentiating 3R (exclusion) and 4R (inclusion) tau isoforms. Tauopathies, a group of neurodegenerative disorders characterized by tau aggregation, follow a similar classification (3R, 4R) based on the predominance of a specific tau isoform in the pathogenic aggregates that define each pathology. As such, disorders such as CBD and PSP are classified as 4R tauopathies, while PiD represents a 3R tauopathy. Interestingly, the majority of familial, rare pathogenic variants identified have been shown to be associated with phenotypes consistent with PSP, CBD or globular glial tauopathy, as well as an increase in either 4R or 3R + 4R tau expression.^15^ Less frequently, familial *MAPT* variants have been linked to Pick-like 3R pathology. Similarly, there have been no rare, intermediate-effect-size, disease-modifying variants identified for PiD.

We aimed to assess the frequency of the rare disease-modifying variant p.A152T in PiD utilizing the large collection of samples available through the Pick’s disease international consortium (PIC). PiD is a subtype of FTLD-tau disorders that clinically presents with behavioural variant frontotemporal dementia (bvFTD), primary progressive aphasia (PPA), AD dementia or corticobasal syndrome (CBS) and is neuropathologically defined by 3R tau inclusions known as Pick bodies.^6,16^ A PiD diagnosis can only be established postmortem upon neuropathological examination of brain tissue, as no biomarkers or clinical diagnostic tools currently exist to allow for an antemortem diagnosis.^16^ As PiD remains understudied due to its rarity, gaining insight into the genetic architecture of the disease is imperative to provide insight into the disease mechanisms and promote future research studies and biomarker development.

## Materials and methods

### Subjects

We utilized pathology-confirmed PiD cases from the PIC to screen for the MAPT p.A152T variant. All PiD samples included in our study were assessed by a neuropathologist according to the published operational criteria for PiD.^6^ In total, we included 401 PiD cases (Table 1; Supplementary Table 1). As a comparison group, we also utilized previously published control data from our laboratory.^17^ In total, we analysed *N* = 2456 clinically evaluated healthy control individuals. Controls were defined as participants who were free of symptoms of any neurodegenerative disorder at the time of clinical examination. This study was approved by the Mayo Clinic Institutional Review Board, and the patient/next of kin provided signed consent for participation in neurodegenerative disease research. The work described has been carried out in accordance with The Code of Ethics of the World Medical Association (Declaration of Helsinki) for experiments involving humans.

**Table 1:** Summary of characteristics of the individuals with PiD and control samples included in the genetic screening for MAPT p.A152T

	PiD		Controls^a^	
Variable	*N*	Participants	*N*	Participants
**Age, years**	394	69 (40, 100)	2456	71 (18, 100)
**Age at disease onset, years**	346	58 (33, 80)	N/A	N/A
**Disease duration, years**	344	10 (2, 25)	N/A	N/A
**Sex (female)**	395	157 (40%)	2456	1350 (55%)

Age refers to age at death for the PiD cases, while age at collection is reported for the control samples.

N/A = not applicable.

^a^Control data was previously published^17^.

### MAPT p.A152T screening

DNA was extracted from frozen brain tissue using standard methods. Samples from North American sites (*n* = 257) were screened with a genotyping probe designed to rs143624519 using an ABI TaqMan™ allelic discrimination assay on the QuantStudio™ 7 Flex Real-Time PCR System (Thermo Fisher Scientific, Waltham, MA) and further analysed in the QuantStudio software. Samples from European sites (*n* = 144) were genotyped using Single Nucleotide Polymorphism (SNP) array data generated by the Illumina NeuroBooster Array. Genotypes were called using GenomeStudio version 2.0 (Illumina).Variant frequency was also evaluated in 2456 healthy control samples, including both clinical samples and neuropathologically healthy brains, using TaqMan data previously generated by our group following the protocol described above.^17^

Bi-direction Sanger sequencing was performed on exon 7 of the *MAPT* gene to confirm identified mutation carriers using the Applied Biosystems DNA Analyzer 3730xl. All sequences were analysed and visualized using SeqScape Software v3.0 (Thermo Fisher Scientific, Waltham, MA, USA).

### Statistical analysis

The association of presence of the minor allele of the MAPT p.A152T variant with risk of PiD (i.e. versus controls) was assessed using a logistic regression model that was adjusted for sex and age (age at death in PiD subjects, age at sample collection in controls). Odds ratio (OR) and 95% confidence interval (CI) were estimated. Statistical analyses were performed using R Statistical Software (version 4.2.2).

### Bulk RNA Sequencing on MAPT p.A152T carriers

To assess the potential impact of the MAPT p.A152T variant on *MAPT* transcript expression, we utilized short-read, bulk RNA Sequencing (RNA-Seq) data generated for *n* = 12 AD cases [*n* = 7 mutation carriers (MT), *n* = 5 wild-type (WT)] and *n* = 8 PSP cases (*n* = 5 mutation carriers, *n* = 3 WT) (Table 2).

**Table 2:** Summary of characteristics of individuals with AD and PSP included in bulk RNA-Seq per MAPT p.A152T genotype

	Median (minimum, maximum) or no. (%)			
	AD (*N* = 12)		PSP (*N* = 8)	
	MAPT p.A152T carriers (*n* = 7)	WT (*n* = 5)	MAPT p.A152T carriers (*n* = 5)	WT (*n* = 3)
**Sex (female)**	4 (57%)	3 (60%)	3 (60%)	1 (33.3%)
**Age at death (yr)**	81 (67, 96)	83 (80, 91)	77 (67, 81)	76 (69, 80)
**RIN**	9.4 (4.6, 9.9)	9.6 (7.4, 9.8)	9.3 (9.2, 9.9)	9.7 (9.3, 9.8)

WT = wild-type.

RNA was extracted from the frontal cortex of frozen human brain tissue using standard protocols. RNA integrity was assessed using the Agilent 2100 Bioanalyzer RNA Nano Chip (median RNA Integrity Number = 9.45). Library preparation and RNA-Seq were performed at the Mayo Clinic Genome Analysis Core in Rochester, MN, using the Illumina Access library prep. Samples were sequenced across two lanes of the NovaSeq S4 PE100 (Illumina).

Upon sequencing, compressed FASTQ files containing short RNA-Seq raw reads were used to run kallisto, a program using pseudoalignment to provide transcript-level information from bulk short-read RNA-Seq data.^18^ First, using FASTQ, we run kallisto quant, which uses a pre-built transcriptome index based on ENCODE version 47 annotations to quantify transcript abundances from the input reads. kallisto h5dump was then used to process the kallisto quant output and generate an abundance.tsv file, which included both estimated counts and TPM (transcripts per million) values for each transcript. Expression values for all identified *MAPT* transcripts were extracted. Identified transcripts were annotated based on the presence of exon 10 as 3R and 4R transcripts using the appropriate sequences available through Ensembl. Furthermore, Ensembl was used to annotate samples per Biotype [protein coding, nonsense-mediated decay (NMD), no protein]. Transcripts that are predicted to result in NMD or non-coding products were excluded from downstream analysis. Transcripts were then grouped based on their 3R/4R classification, and TPM values were added to obtain 3R, 4R and total tau counts per sample. These values were also used to calculate 4R/3R ratios per sample. Statistical analysis was then performed using R Statistical Software (version 4.2.2). Specifically, we assessed differences in 3R and 4R *MAPT* transcript expression as well as 4R/3R ratios using total TPM count values between mutation carriers and WT samples within each disease group using unadjusted linear regression models. Though the ability to perform multivariable analysis adjusting for confounding variables is limiting owing to the relatively small sample sizes, in a sensitivity analysis, we also adjusted these models for sex and age at death and observed similar findings. Protein coding *MAPT* transcripts were plotted using the gff3 file available in Ensembl and ggtranscript.^19^

## Results

### MAPT p.A152T frequency in Pick’s disease

We screened 401 PiD samples from the PIC for the MAPT p.A152T risk variant. MAPT p.A152T (rs143624519) is a missense variant located within exon 7 (Chr17:45991484 G > A) of the *MAPT* gene. Within the PiD cohort, we identified one heterozygous mutation carrier [minor allele frequency (MAF) = 0.12%], which was further confirmed via Sanger sequencing. The single individual identified as the heterozygous carrier was a non-Hispanic/Latino female who presented with a clinical diagnosis of AD and died at 66 years of age (average age of death in the cohort was 69; Table 1). Genomic analysis demonstrated that she carried the *MAPT* H1/H1 haplotype and the *APOE* ε3/ε3 genotype. The MAF for MAPT p.A152T in PiD was slightly lower than the MAF of 0.20% in the 2456 controls (10 carriers); however, this difference did not approach statistical significance (OR 0.69, 95% CI 0.09–5.50, *P* = 0.73). Interestingly, the MAF of MAPT p.A152T is lower in PiD compared to the MAF reported for FTD spectrum disorders and AD (0.44% and 0.34% respectively).^12^

### Effect of MAPT p.A152T on MAPT transcript expression

**Figure 1:**
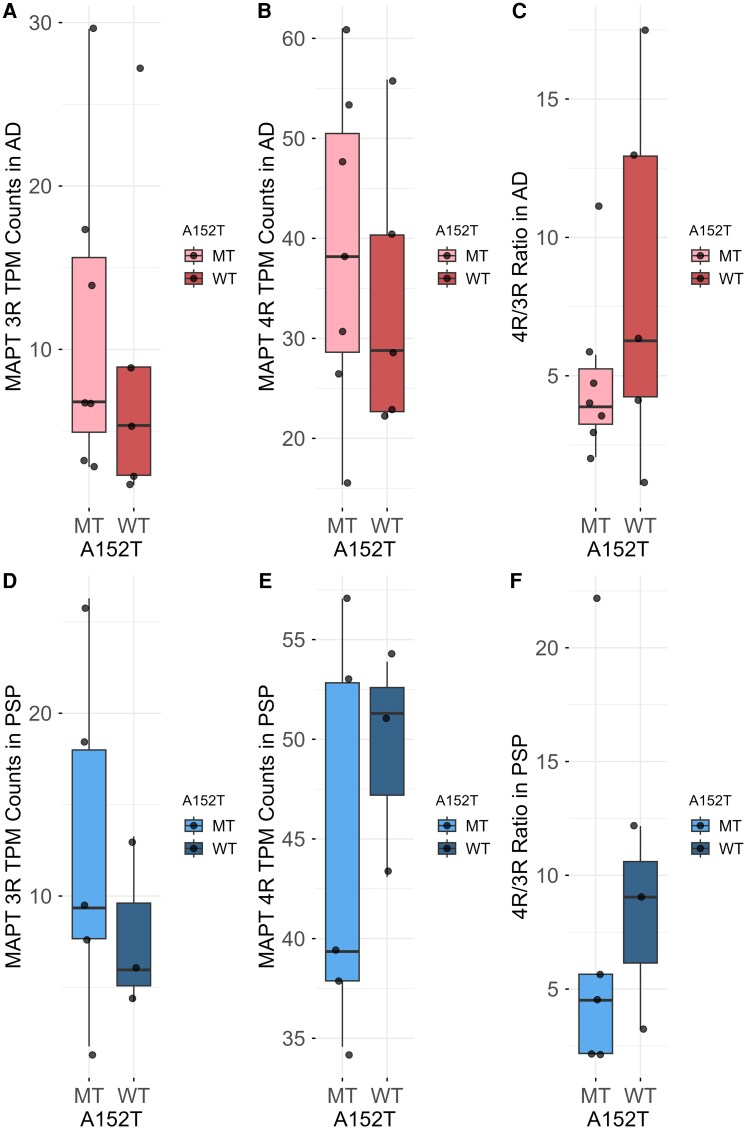
Overview of *MAPT* 3R and 4R transcript expression and 4R/3R *MAPT* ratios in AD (*n* = 12) and PSP (*n* = 8) samples stratified by MAPT p.A152T status. Associations were assessed using linear regression models, both unadjusted and adjusted for sex and age at death. (**A**) *MAPT* 3R transcript expression in AD samples. (**B**) *MAPT* 4R transcript expression in AD samples. (**C**) 4R/3R *MAPT* ratio in AD samples. (**D**) *MAPT* 3R transcript expression in PSP samples. (**E**) MAPT 4R transcript expression in PSP samples. (**F**) 4R/3R *MAPT* ratio in PSP samples. MT = MAPT p.A152T mutation carriers; WT = wild-type genotype; TPM = transcript per million. No significant associations were observed, including exclusion of extreme outliers. Individual points represent individual samples.

Given that MAPT p.A152T is a risk factor for both AD and PSP, two tauopathies defined by 4R tau aggregation, but does not seem to influence the risk for PiD, it is possible that it does not have an effect on 3R tau pathology. Instead, MAPT p.A152T may preferentially influence 4R tau expression at the transcript level, ultimately leading to increased levels and subsequent aggregation of 4R tau protein isoforms. As such, to understand the potential effects of MAPT p.A152T on *MAPT* transcript-specific expression, we utilized bulk RNA-Seq data from the frontal cortex tissue of mutation carriers and WT individuals with PSP and AD pathologies (Table 2). Using kallisto, we were able to identify 22 *MAPT* transcripts expressed in our cohort (Supplementary Table 2). Of those, 15 transcripts were annotated as non-protein coding or NMD and were excluded from further analysis (Supplementary Table 2). Within the remaining seven protein-coding transcripts, we were able to detect two 3R transcripts and five 4R transcripts, including several non-canonical transcripts, such as big-tau (exon 4a) isoforms (Supplementary Fig. 1). We used these to assess total *MAPT* expression and observed that total tau expression was similar between mutation carriers and WT AD and PSP samples. Then, we explored expression patterns of 3R and 4R *MAPT* protein-coding transcripts as well as their ratios; however, we did not detect any significant changes in expression levels between WT and MT samples in any of the groups (Fig. 1). We further examined N-terminal protein-coding transcripts and observed no statistically significant differences across groups. Initial trends indicate that AD mutation carriers have higher levels of 2N4R transcripts compared to WT cases, whereas PSP mutation carriers exhibited increased 1N3R and decreased 0N4R transcript levels relative to WT cases (Supplementary Fig. 2).

## Discussion

Genetic studies on neurodegenerative disorders, and particularly tauopathies, have provided important insight into disease mechanisms. Variants in the *MAPT* gene have been shown to contribute to tauopathy pathogenesis and increased disease risk. A subset of these variants are highly penetrant mutations that were found to segregate with disease in families.^7^ However, the rare MAPT p.A152T variant, which has been identified as a risk factor for AD and FTD spectrum disorders, has not been observed to segregate within families. We therefore sought to investigate whether this variant might also be associated with the risk for another sporadic FTD-spectrum disorder, PiD. PiD is a rare 3R tauopathy for which no familial cases have been reported, and to date, no highly penetrant, disease-causing mutations have been identified. Nonetheless, it is possible that rare variants contributing to the risk of other tauopathies may also play a role in PiD susceptibility. In this study, we examined the prevalence of the MAPT p.A152T variant in the largest series of neuropathologically confirmed PiD cases assembled to date.

We screened 401 PiD cases, and although no significant association was observed, we note a lower frequency of the MAPT p.A152T variant in PiD cases compared to what was previously reported in control samples.^17^ Given the previous risk association in 4R tauopathies,^12-14^ this could indicate that the MAPT p.A152T variant contributes to disease through an increase in expression and subsequent production and accumulation of 4R tau. To test whether the variant might have an effect at the isoform level, we used bulk RNA-Seq data from PSP and AD WT and MAPT p.A152T carriers and obtained *MAPT* transcript-level expression data for these cases. Interestingly, we did not observe any significant differences in 4R tau expression between MT and WT samples in either disease group. Although our limited sample size reduces the power to detect statistically significant associations, the complexity of tau transcript expression underscores the need for additional studies. Further studies should also be conducted to directly compare MAPT p.A152T variant frequencies in pathologically confirmed control, PiD, AD and PSP cohorts, as prior investigations have relied on mixtures of clinically and pathologically classified cases when calculating variant frequencies.

Overall, our findings do not support a clear association between the MAPT p.A152T variant and increased 4R tau transcript levels in the human brain. It is possible that there are more nuanced effects on transcription, such as a preferential increase of specific N-terminal transcripts or the effect on 4R tau aggregation and clearance. While we began to elucidate some of those trends, short-read bulk RNA-Seq and tools relying on pseudoalignments approaches, such as kallisto, are not the preferred methods to provide accurate full-length transcript information.^20^ As such, employing long-read RNA-Seq on these cases in the future will be imperative in delineating whether the MAPT p.A152T mutation preferentially affects specific *MAPT* transcripts and provide information on any potentially novel splice variants. Future studies should also explore cell-type localization of transcripts as it is possible that the variant acts through a specific cell-type pathway to differentially mediate disease in the tauopathies.

It is also important to consider other potential mechanisms by which the MAPT p.A152T variant could be acting to modify disease. A previous study using *Caenorhabditis elegans* and mouse models has suggested that the variant is associated with impaired retrograde axonal transport,^21^ while another study showed that the MAPT p.A152T variant leads to tau-dependent neuronal hyperactivity in Induced Pluripotent Stem Cells (iPSC)-derived neurons.^22^ The variant is located within exon 7 of the *MAPT* gene, a constitutive spliced exon that encodes for part of the proline-rich domain of the tau protein.^23^ This domain has been shown to be involved in signal transduction through the interaction with PLC-γ isoenzymes and src-family tyrosine kinases as well as tau associations with actin and tubulin.^24-26^ More recently, studies have begun to suggest the importance of the tau proline-rich domain in disease as it is a region with high frequency of phosphorylation sites, with the phosphorylation of the neighbouring T153 residue being implicated in tauopathy risk in mouse studies.^27,28^ Interestingly, increased levels of soluble pT153-positive tau from postmortem human brain were shown to differentiate A152T carriers from noncarriers in AD, PSP and CBD.^28^

In conclusion, the MAPT p.A152T risk variant, previously described in PSP and AD patients, does not seem to influence the risk of 3R primary tauopathy PiD. It is thus possible that it is linked to an increased risk of 4R or mixed (3R + 4R) pathologies. Previous studies have demonstrated that the variant might influence various pathways, from phosphorylation of the neighbouring T153 residue and altered solubility profiles to neuronal firing and axonal transport. However, its exact mechanism of action remains elusive and might differ across the tauopathies. While highlighting the rarity of the variant, focusing future functional studies on specific pathologies might help elucidate the variant’s mechanism of action across the tauopathy spectrum and aid in the development of accurate disease-specific therapeutic approaches.

## Supplementary Material

fcag266_Supplementary_Data

## Data Availability

The data supporting the findings of this study are available within the manuscript and its supplementary material.

## Appendix

PIC members

Rebecca R Valentino Ph.D.^1^, Tammaryn Lashley Ph.D.^2,3^, Hannah L Macpherson M.Sc.^3^, Thomas T Warner Ph.D., FRCP^2,4^, Zane Jaunmuktane M.D., FRCPath^2,4^, Alejandro Martinez-Carrasco M.Sc.^4^, Kin Mok Ph.D., FRCP^3,5,6,7^, Tamas Revesz Ph.D., FRCPath^2,3^, Elizabeth A Christopher M.B.A.^1^, Michael DeTure Ph.D.^1^, Shunsuke Koga M.D., Ph.D.^1^, Neill R Graff-Radford M.D.^8^, Bradley F Boeve M.D.^9^, Keith A Josephs M.D.^9^, Ronald C Petersen M.D., Ph.D.^9^, Jennifer Whitwell Ph.D.^10^, Ranjan Duara M.D.^11^, Kelly E Lyons Ph.D.^12^, Rajesh Pahwa M.D.^12^, Lea T Grinberg M.D., Ph.D.^13^, William W Seeley M.D.^13^, Bruce Miller M.D.^13^, Athena Schlereth ^13^, Salvatore Spina M.D., Ph.D.^13^, David J Irwin M.D.^14^, David A Wolk M.D.^14^, EunRan Suh Ph.D.^15^, Vivianna M Van Deerlin M.D. Ph.D.^15^, Edward B Lee M.D., Ph.D.^15^, Matthew P Frosch M.D., Ph.D.^16^, Theresa R Connors B.S.^16^, Derek H Oakley M.D.^16^, Laura Molina-Porcel M.D., Ph.D.^17,18,19^, Iban Aldecoa M.D., Ph.D.^17,20,21^, Mircea Balasa M.D., Ph.D.^18,19^, Sergi Borrego-Ecija M.D.^18,19,21^, Raquel Sanchez-Valle M.D., Ph.D.^18,19,21^, Jordi Gascon-Bayarri M.D.^22^, Pilar Sanz-Cartagena M.D., Ph.D.^23^, Gerard Pinol-Ripoll M.D., Ph.D.^24^, Rosa Maria de Eugenio Huélamo M.D.^25^, Ellen Gelpi M.D., Ph.D.^26^, Tamar Gefen Ph.D.^27,28^, Rudolph J Castellani M.D.^29^, Margaret E Flanagan M.D.^27,29,30,31^, Emily J Rogalski ^27,28,32,33^, Sandra Weintraub Ph.D.^27,28^, Julie A. Schneider M.D., M.S.^34^, Kevin F Bieniek Ph.D.^30,31^, Xiongwei Zhu Ph.D.^35^, Javier Redding-Ochoa M.D.^36^, Koping Chang M.D.^36^, Juan C Troncoso M.D.^36^, Stefan Prokop M.D.^37^, Bernardino Ghetti M.D.^38^, Kathy L Newell M.D.^38^, Max Jacobsen B.S.^38^, Andrew C Robinson Ph.D.^39,40^, Federico Roncaroli M.D.^39,40^, Christopher Kobylecki PhD, FRCP^41,42^, Matthew Jones M.D., FRCP^41,42^, Anna Richardson B.Sc., FRCP^41,42^, Julie Snowden Ph.D.^41,42^, James B Rowe Ph.D.^43,44^, Annelies Quaegebeur M.D., Ph.D.^45^, Kieren Allinson FRCPath^46^, Thomas G Beach M.D., Ph.D.^47^, Geidy E Serrano Ph.D.^47^, Andrew F Teich M.D., Ph.D.^48,49^, Xena E Flowers B.S.^49^, Allison C Heaps M.S.^49^, Sandra P Leskinen M.A.^49^, Julia L Keith M.D., FRCPC^51^, Sandra E Black M.D., FRCPC^51^, Mario Masellis M.Sc., M.D., Ph.D., FRCPC^51^, Andrew King FRCPath^52,53^, Safa-Al Sarraj FRCPath^52,53^, Claire Troakes Ph.D.^53^, Glenda M Halliday Ph.D.^54^, John R Hodges M.D.^54^, Jillian J Kril Ph.D.^54^, John B Kwok Ph.D.^54^, Olivier Piguet Ph.D.^55^, Marla Gearing Ph.D.^56,57,58^, Sigrun Roeber M.D.^59^, Christopher M Morris Ph.D.^61^, Johannes Attems M.D.^61^, Alan J Thomas Ph.D.^61^, Charles L White, 3rd M.D.^62^, Bret M Evers M.D., Ph.D.^62^, Naguib Mechawar Ph.D^63^, Susana Boluda MD^64,65^, Danielle Seilhean M.D., Ph.D.^66^, Isabelle Le Ber M.D., Ph.D.^67,68^, Sabrina Turbant-Leclere Ph.D.^67^, Ian R MacKenzie M.D.^69^, Anne A Sieben MD, Ph.D.^70,71,72,73^, Patrick P Cras MD, Ph.D.^70,71,73^, Bart B De Vil M.Sc. Eng.^70,71,73^, P.P. De Deyn MD, Ph.D.^74^, C. Dirk Keene MD, PhD^75^, Caitlin S Latimer MD, PhD^75^, Aimee Schantz MS^75^, Thomas D Bird MD^76,77,78^, Vahram Haroutunian Ph.D.^79^, Dushyant Purohit M.D.^79^, Jamie M Walker M.D., Ph.D.^79^, Robert Rissman Ph.D.^81^, Catriona McLean M.D., Ph.D.^82,83^, Matthew D Cykowski M.D.^84^, Shih-Hsiu J Wang M.D., Ph.D.^84^, John F Ervin ^85^, Caroline Graff Ph.D.^86,87^, Inger Nennesmo M.D.^88,89^, Rashed M Nagra Ph.D.^90^, James Riehl B.S.^91^, Gabor G Kovacs M.D., Ph.D.^92,93,94^, Giorgio Giaccone M.D.^95^, Manuela Neumann M.D.^98,99^, Lee-Cyn Ang M.D.^100,101^, Elizabeth C Finger M.D.^101,102^, Agostinho Carvalho Ph.D.^103,104^, Cristina Cunha Ph.D.^103,104^, Huw R Morris Ph.D., FRCP^105^, Cornelis Blauwendraat Ph.D.^106^, Mike A Nalls Ph.D.^106,107,108^, Andrew B Singleton Ph.D.^106^, Dan Vitale M.Sc.^106,107,108^, Richard J Perrin M.D., Ph.D.^109,110^, Anne M Chaffee P.A. (ASCP)^109^, Zbigniew K Wszolek M.D.^8^.

^1^Department of Neuroscience, Mayo Clinic, Jacksonville, FL 32224, USA; ^2^Queen Square Brain Bank for Neurological Disorders, University College London, Queen Square Institute of Neurology, London, UK; ^3^Department of Neurodegenerative Disease, University College London, Queen Square Institute of Neurology, London, UK; ^4^Department of Clinical and Movement Neurosciences, University College London, Queen Square Institute of Neurology, London, UK; ^5^UK Dementia Research Institute at UCL, London, UK; ^6^Division of Life Science, State Key Laboratory of Molecular Neuroscience, Molecular Neuroscience Center, The Hong Kong University of Science and Technology, Clear Water Bay, Kowloon, Hong Kong, China; ^7^Hong Kong Center for Neurodegenerative Diseases, Hong Kong Science Park, Hong Kong, China; ^8^Department of Neurology, Mayo Clinic, Jacksonville, FL 32224, USA; ^9^Department of Neurology, Mayo Clinic, Rochester, MN 55905, USA; ^10^Department of Radiology, Mayo Clinic, Rochester, MN 55905, USA; ^11^Wien Center for Alzheimer’s Disease and Memory Disorders, Mount Sinai Medical Center, Miami Beach, FL; ^12^University of Kansas Medical Center, Parkinson’s Disease & Movement Disorder Division, Kansas City, KS 66160; ^13^Department of Neurology, Memory and Aging Center, University of California San Francisco, San Francisco, CA, USA; ^14^Department of Neurology, Perelman School of Medicine at the University of Pennsylvania, Philadelphia, PA 19104, USA; ^15^Department of Pathology and Laboratory Medicine, Perelman School of Medicine at the University of Pennsylvania, Philadelphia, PA 19104, USA; ^16^Neuropathology Service, C.S. Kubik Laboratory for Neuropathology, Massachusetts General Hospital/Harvard Medical School, Boston, MA, USA; ^17^Neurological Tissue Bank, Biobanc-Hospital Clínic-Fundació de Recerca Clínic Barcelona-Institut d’Investigacions Biomèdiques August Pi i Sunyer, Barcelona, Spain; ^18^Alzheimer’s Disease and other Cognitive Disorders Unit, Neurology Department, Hospital Clinic, Barcelona, Spain; ^19^Barcelona Clinical Research Foundation-August Pi i Sunyer Biomedical Research Institute, Barcelona, Spain; ^20^Pathology Department, Biomedical Diagnostic Center, Hospital Clinic de Barcelona-University of Barcelona, Barcelona, Spain; ^21^University of Barcelona, Barcelona, Spain; ^22^Servei de Neurologia, Hospital Universitari de Bellvitge, Institut d'Investigació Biomèdica de Bellvitge (Idibell), L’Hospitalet de Llobregat, Spain; ^23^Hospital de Mataro, Carr. de Cirera, 230, 08304 Mataró, Barcelona, Spain; ^24^Unitat Trastorns Cognitius (Cognitive Disorders Unit), Clinical Neuroscience Research, IRBLleida, Santa Maria University Hospital, Lleida, Spain; ^25^Hospital de Palamós, Carrer Hospital, 36, 17230 Palamós, Girona, Spain; ^26^Division of Neuropathology and Neurochemistry, Department of Neurology, Medical University of Vienna, Vienna, Austria; ^27^Mesulam Center for Cognitive Neurology and Alzheimer’s Disease, Northwestern University Feinberg School of Medicine, Chicago, IL, USA; ^28^Department of Psychiatry and Behavioral Sciences, Northwestern University Feinberg School of Medicine, Chicago, IL, USA; ^29^Department of Pathology, Northwestern University Feinberg School of Medicine, Chicago, IL, USA; ^30^Glenn Biggs Institute for Alzheimer’s and Neurodegenerative Diseases, San Antonio, TX, USA; ^31^Department of Pathology and Laboratory Medicine, University of Texas Health San Antonio, San Antonio, TX, USA; ^32^Healthy Aging & Alzheimer’s Research Care Center, Biological Sciences Division, University of Chicago, IL; ^33^Department of Neurology, Biological Sciences Division, University of Chicago, IL; ^34^Rush Alzheimer’s Disease Center, Rush University Medical Center, Chicago, IL, USA; ^35^Department of Pathology, Case Western Reserve University, 2103 Cornell Road, Cleveland, OH 44106, USA; ^36^Johns Hopkins School of Medicine, Baltimore, MD, USA; ^37^Fixel Institute for Neurological Diseases, University of Florida, Gainesville, FL, USA; ^38^Department of Pathology and Laboratory Medicine, Indiana University School of Medicine, Indianapolis, IN, USA; ^39^Division of Neuroscience, Faculty of Biology, Medicine and Health, School of Biological Sciences, The University of Manchester, Salford Royal Hospital, Salford M6 8HD, UK; ^40^Geoffrey Jefferson Brain Research Centre, Manchester Academic Health Science Centre (MAHSC), Manchester, UK; ^41^Department of Neurology, Manchester Centre for Clinical Neurosciences, Northern Care Alliance NHS Foundation Trust, Manchester Academic Health Science Centre, University of Manchester, Manchester, UK; ^42^Division of Neuroscience, School of Biological Sciences, University of Manchester, Manchester, UK; ^43^Cambridge University Department of Clinical Neurosciences and Cambridge University Hospitals NHS Trust, Cambridge, UK; ^44^Medical Research Council Cognition and Brain Sciences Unit, Cambridge, UK; ^45^Department of Histopathology, Cambridge University Hospitals NHS Foundation Trust, Cambridge, UK; ^46^Histopathology Box 235 Cambridge University Hospital NHS Foundation Trust, Cambridge Biomedical Campus, Hills Road, Cambridge, CB2 0QQ; ^47^Civin Laboratory of Neuropathology, Banner Sun Health Research Institute, Sun City, AZ, USA; ^48^Department of Pathology and Cell Biology, Columbia University, New York, NY, USA; ^49^Taub Institute for Research on Alzheimer’s Disease and the Aging Brain, Columbia University, New York, NY, USA; ^49^Taub Institute for Research on Alzheimer’s Disease and the Aging Brain, Columbia University, New York, NY, USA; ^51^Laboratory Medicine and Molecular Diagnostics, Sunnybrook Health Sciences Centre, and Laboratory Medicine and Pathobiology, University of Toronto, Toronto, Canada; ^52^Clinical Neuropathology Department, King’s College Hospital NHS Foundation Trust, London, UK; ^53^London Neurodegenerative Diseases Brain Bank, Department of Basic and Clinical Neuroscience, Institute of Psychiatry, Psychology and Neuroscience, King's College London, London, UK; ^54^University of Sydney Brain and Mind Centre and Faculty of Medicine and Health School of Medical Sciences, Camperdown, NSW, Australia; ^55^University of Sydney Brain and Mind Centre and Faculty of Science School of Psychology; ^56^Dept. of Pathology and Laboratory Medicine, Emory University School of Medicine, Atlanta, GA, USA; ^57^Dept. of Neurology, Emory University School of Medicine, Atlanta, GA, USA; ^58^Goizueta Alzheimer's Disease Center Brain Bank, Emory University School of Medicine, Atlanta, GA USA; ^59^Center for Neuropathology and Prion Research, Ludwig-Maximilians-University Munich, Germany; ^60^Department of Psychiatry and Psychotherapy, University Hospital, Ludwig-Maximilians-University Munich, Germany; ^61^Newcastle Brain Tissue Resource, Translational and Clinical Research Institute, Newcastle University, Newcastle upon Tyne, NE4 5PL, UK; ^62^University of Texas Southwestern Medical Center, Dallas, TX, USA; ^63^Douglas Hospital Research Centre, McGill University, Montreal, QC, Canada; ^64^Department of Neuropathology, AP-HP, Hôpital de la Pitié Salpêtrière, DMU Neuroscience, Paris, France; ^65^Sorbonne University, Paris Brain Institute-ICM, Inserm, CNRS, AP-HP, Hôpital de la Pitié Salpêtrière, Paris, France; ^66^Laboratoire de Neuropathologie Escourolle, Hôpital de la Salpêtrière, Assistance Publique–Hôpitaux de Paris, Paris, France; ^67^Inserm U1127, CNRS UMR 7225, Sorbonne Université, Paris Brain Institute (ICM), Hôpital Pitié-Salpêtrière, Paris, France; ^68^Centre de référence des démences rares ou précoces, Hôpital Pitié-Salpêtrière, Paris, France; ^69^Department of Pathology and Laboratory Medicine, University of British Columbia, Vancouver, BC, Canada; ^70^Laboratory of Neurology, Translational Neurosciences, Faculty of Medicine and Health Sciences, University of Antwerp, Antwerp, Belgium; ^71^IBB-NeuroBiobank BB190113, Born Bunge Institute, Antwerp, Belgium; ^72^Department of Pathology, Antwerp University Hospital, Antwerp, Belgium; ^73^Department of Neurology, Ghent University Hospital, Ghent University, Belgium; ^74^Laboratory of Neurochemistry and Behavior, Experimental Neurobiology Unit, University of Antwerp, Universiteitsplein 1, 2610 Antwerpen, Belgium; ^75^Department of Laboratory Medicine and Pathology, University of Washington School of Medicine; ^76^Geriatrics Research Education and Clinical Center, Veterans Affairs Puget Sound Health Care System, Seattle, WA, USA; ^77^Department of Neurology, University of Washington, Seattle, Washington, USA; ^78^Division of Medical Genetics, Department of Medicine, University of Washington, Seattle, WA, USA; ^79^Icahn School of Medicine at Mount Sinai; ^80^University of California, San Diego, School of Medicine; ^81^Alzheimer’s Therapeutic Research Institute, Keck School of Medicine of the University of Southern California, San Diego, California, USA; ^82^Department of Anatomical Pathology Alfred Heath, Melbourne, Victoria, 3004, Australia; ^83^Victorian Brain Bank, The Florey Institute of Neuroscience of Mental Health, Parkville, Victoria, 3052, Australia; ^84^Department of Pathology and Genomic Medicine, Houston Methodist Research Institute and Weill Cornell Medicine, Houston, TX, USA; ^85^Department of Neurology, Duke University Medical Center, Durham, USA; ^86^Division for Neurogeriatrics, Centre for Alzheimer Research, Department of Neurobiology, Care Sciences and Society, Karolinska Institutet, Stockholm, Sweden; ^87^Unit for Hereditary Dementias, Karolinska University Hospital Solna, Stockholm, Sweden; ^88^Department of Oncology-Pathology, Karolinska Institutet, Stockholm, Sweden; ^89^Department of Pathology and Cancer Diagnostics, Karolinska University Hospital Solna, Stockholm, Sweden; ^90^Human Brain and Spinal Fluid Resource Center, Brentwood Biomedical Research Institute, Los Angeles, CA, United States; ^91^UCLA—Sepulveda, Los Angeles, CA; ^92^Tanz Centre for Research in Neurodegenerative Disease (CRND), University of Toronto, Toronto, ON, Canada; ^93^Department of Laboratory Medicine and Pathobiology, University of Toronto, Toronto, ON, Canada; ^94^Laboratory Medicine Program and Krembil Brain Institute, University Health Network, Toronto, ON, Canada; ^95^Fondazione Istituto di Ricovero e Cura a Carattere Scientifico, Istituto Neurologico Carlo Besta, Milan, Italy; ^96^Department of Neuroscience, Psychology, Drug Research and Child Health University of Florence, Florence, Italy; ^97^IRCCS Fondazione Don Carlo Gnocchi, Florence, Italy; ^98^Molecular Neuropathology of Neurodegenerative Diseases, German Center for Neurodegenerative Diseases (DZNE), Tübingen, Germany; ^99^Department of Neuropathology, University Hospital of Tübingen, Tübingen, Germany; ^100^Department of Pathology and Laboratory Medicine, London Health Sciences Centre, London, ON, Canada; ^101^Schulich School of Medicine and Dentistry, Western University, London, ON, Canada; ^102^Department of Clinical Neurological Sciences, Western University, London, ON, Canada; ^103^Life and Health Sciences Research Institute (ICVS), School of Medicine, University of Minho, Braga, Portugal; ^104^ICVS/3B’s—PT Government Associate Laboratory, Braga/Guimarães, Portugal; ^105^Department of Clinical and Movement Neurosciences, University College London, Queen Square Institute of Neurology London, UK; ^106^Laboratory of Neurogenetics, National Institute on Aging, National Institutes of Health, Bethesda, MD, USA; ^107^Center for Alzheimer’s and Related Dementias, National Institutes of Health, Bethesda, MD, USA; ^108^Data Tecnica International LLC, Washington, DC, USA; ^109^Department of Pathology and Immunology, Washington University School of Medicine, Saint Louis, Missouri, 63110; ^110^Department of Neurology, Washington University School of Medicine, Saint Louis, Missouri, 63110.
