# Supplementary material for: Evaluating MAPT p.A152T as a risk factor for the 3R tauopathy Pick’s disease
Source: Brain Commun. 2026 Jul 9;8(4):fcag266. doi: 10.1093/braincomms/fcag266 (PMC13435620; doi:10.1093/braincomms/fcag266)
Supplement: Supplementary file 1 [file fcag266_supplementary_data.pdf]

Evaluating MAPT p.A152T as a risk factor for the 3R tauopathy Pick's disease

Supplementary Material

Institution	Location	Number of cases genotyped
Banner Sun Health Research	Sun City, AZ, USA	9
Cambridge Brain Bank	Cambridge, UK	10
Case Western Reserve University	Cleveland, OH, USA	4
Columbia University	New York City, NY, USA	9
Douglas-Bell Canada Brain Bank	Montreal, QC, Canada	3
Duke School of Medicine	Durham, NC, USA	3
DZNE	Göttingen, Germany	1
Emory University	Atlanta, GA, USA	7
Houston Methodist Hospital	Houston, TX, USA	2
IDIBAPS	Barcelona, Spain	14
Indiana University School of Medicine	Indianapolis, IN, USA	12
KCL (London Neurodegenerative Diseases Brain Bank)	London, UK	8
IRCCS Foundtion Carlo Besta Neurological Institute	Milan, Italy	2
John Hopkins University	Baltimore, MD, USA	13
Karolinska	Solna, Sweden	3
Krembil Research Institute, University of Toronto	Toronto, ON, Canada	2
Manchester Brain Bank	Manchester, UK	11
Massachusetts General Hospital	Boston, MA, USA	15
Mayo Clinic Jacksonville	Jacksonville, FL USA	70
Mayo Clinic Rochester	Rochester, MN, USA	5
Mount Sinai Neuropathology Brain Bank	New York City, NY, USA	3
Neurobiobak Munchen	Munich, Germany	7

Netherlands Brain Bank	Amsterdam, Holland	33
Neuro-CEB	Paris, France	5
Newcastle Brain Bank	Newcastle, UK	7
Northwestern University	Chicago, IL, USA	14
Oxford Brain Bank	Oxford, UK	7
Parkwood Institute, University of Western Ontario	London, ON, Canada	1
Rush Alzheimer's Disease Center, Rush University	Chicago, IL, USA	8
South West Brain Bank	Bristol, UK	8
Sunnybrook Health Sciences Centre	Toronto, ON, Canada	9
UBC Hospital	Vancouver, BC, Canada	6
UCL	London, UK	18
UCLA	Los Angeles, CA, USA	1
University of California San Diego	San Diego, CA, USA	9
University of California San Francisco	San Francisco, CA, USA	20
University of Florida	Gainesville, FL, USA	1
University of Pennsylvania	Philadelphia, PA, USA	17
University of Texas Health Science Center at San Antonio	San Antonio, TX, USA	1
University of Washington Neuropathology Core	Seattle, WA, USA	8
University Texas Southwestern	Dallas, TX, USA	7
VBB	Sydney, Australia	5
Washington University	St. Louis, MO, USA	3

Supplementary Table 1. Table of recruitment sites that have contributed Pick's disease cases to the Pick's disease international consortium (PIC) that were genotyped for the MAPT p.A152T variant.

		Sample ID	S1	S2	S3	S4	S5	S6	S7	S8	S9	S10	S11	S12	S13	S14	S15	S16	S17	S18	S19	S20
		**Disease**	AD	PSP	AD	PSP	AD	PSP	AD	PSP	PSP	AD	PSP	AD	AD	PSP	AD	PSP	AD	AD	AD	AD
		**MAPT p.A152T**	MT	MT	WT	MT	WT	MT	WT	MT	WT	MT	WT	MT	WT	MT	MT	WT	MT	WT	MT	MT
		**Age at death**	86	77	80	74	82	81	91	67	69	67	76	91	84	77	80	80	80	83	96	81
		**Sex**	M	M	F	F	F	F	M	M	M	M	M	F	M	F	F	F	F	F	M	F
**Transcript**	**C-terminus**	**Type**																				
ENST00000535772.6	3R	protein-coding	6.79	1.55	8.92	7.67	4.53	9.35	2.30	26.27	5.96	2.81	13.22	17.30	27.24	17.91	13.86	4.22	6.63	1.71	29.59	2.90
ENST00000344290.10	3R	protein-coding	0.00	0.23	0.00	0.00	0.82	0.00	0.00	0.02	0.00	0.00	0.05	0.00	0.02	0.08	0.08	0.00	0.00	0.00	0.02	0.33
ENST00000262410.10	4R	protein-coding	0.10	0.42	0.12	0.00	0.09	0.14	0.20	0.13	0.36	0.10	0.19	0.24	0.11	0.11	1.34	0.53	0.27	0.10	0.22	0.17
ENST00000351559.10	4R	protein-coding	6.02	2.63	6.08	13.85	2.24	4.44	2.53	11.24	5.37	3.73	5.09	9.68	11.33	4.46	7.89	6.99	7.89	2.61	21.89	3.15
ENST00000415613.6	4R	protein-coding	0.00	0.00	0.15	0.00	0.03	0.00	0.00	0.03	0.09	0.13	0.18	0.08	0.00	0.34	0.08	0.00	0.04	0.08	0.09	0.16
ENST00000446361.7	4R	protein-coding	18.94	34.65	47.33	19.22	19.62	45.21	36.93	40.93	43.88	25.53	35.96	40.74	13.27	31.50	35.39	42.13	27.54	17.54	34.40	10.75
ENST00000680674.1	4R	protein-coding	1.29	1.66	2.20	1.50	0.69	3.04	0.67	4.72	4.19	1.39	1.68	2.47	4.08	1.48	3.06	1.65	2.44	1.75	4.41	1.11
ENST00000703922.1	na	nmd	0.37	0.00	0.00	0.54	0.17	0.00	0.00	0.00	0.00	0.00	0.00	0.00	2.92	0.00	0.00	0.00	0.32	0.00	1.47	0.00
ENST00000571311.5	na	nmd	0.15	0.91	0.58	1.80	0.67	1.89	0.13	0.28	0.25	0.55	0.30	0.17	0.11	0.28	0.49	0.31	0.47	1.88	0.28	0.27
ENST00000680542.1	na	nmd	0.03	0.14	0.02	0.00	0.00	0.20	0.04	0.06	0.04	0.11	0.05	0.07	0.04	0.10	0.09	0.00	0.07	0.07	0.06	0.20
ENST00000570299.5	na	no protein	0.01	0.00	0.00	0.01	0.00	0.02	0.00	0.07	0.00	0.00	0.00	0.00	0.01	0.02	0.00	0.00	0.02	0.00	0.04	0.01
ENST00000572440.1	na	no protein	0.75	4.69	3.87	2.20	2.10	6.12	1.55	3.32	3.18	4.27	1.92	3.30	0.85	4.04	5.51	2.65	2.38	3.13	3.62	2.27
ENST00000576518.1	na	no protein	0.10	0.55	0.38	0.29	0.29	0.74	0.11	0.24	0.31	0.50	0.15	0.24	0.11	0.33	0.58	0.21	0.19	0.35	0.30	0.23
ENST00000577017.1	na	no protein	0.30	0.45	1.61	1.41	3.17	0.67	0.10	2.81	3.14	1.11	0.84	1.30	0.77	1.43	2.92	0.04	1.08	0.55	1.67	1.55
ENST00000703974.1	na	no protein	4.87	21.79	40.49	20.60	17.60	27.25	10.94	34.56	27.47	25.07	10.29	22.64	9.84	25.33	33.65	12.65	15.76	10.60	32.61	12.20
ENST00000703975.1	na	no protein	1.38	8.37	2.00	0.70	1.36	10.05	1.73	7.17	3.49	8.01	4.28	6.20	2.12	5.44	4.06	3.42	2.63	7.91	7.45	2.88
ENST00000703976.1	na	no protein	0.35	3.35	7.14	2.76	3.09	3.86	0.70	0.00	3.83	2.04	0.00	0.00	0.00	4.33	7.39	1.08	2.73	0.00	0.00	3.18
ENST00000703977.1	na	no protein	0.50	1.42	1.56	0.15	0.00	2.40	0.16	1.92	0.27	0.94	1.30	1.23	0.38	0.00	0.00	0.13	0.15	0.62	1.63	0.00
ENST00000703979.1	na	no protein	0.00	0.38	0.08	0.00	0.38	0.14	0.06	0.08	0.00	0.07	0.14	0.11	0.06	0.11	0.35	0.12	0.14	0.30	0.19	0.47
ENST00000703980.1	na	no protein	0.00	5.78	3.01	0.00	0.00	18.76	0.00	4.31	13.42	3.26	18.04	13.98	17.83	1.29	0.85	12.73	0.06	3.74	49.65	0.00
ENST00000703981.1	na	no protein	38.94	44.11	71.07	72.06	35.31	33.08	42.79	81.09	51.32	19.61	70.80	87.79	4.19	44.83	57.83	64.39	47.21	7.34	3.88	3.82
ENST00000703982.1	na	no protein	0.27	2.10	3.11	0.48	1.72	3.38	1.04	2.84	2.71	2.44	0.90	2.35	1.01	1.57	2.83	1.41	1.34	0.95	3.54	0.96

Supplementary Table 2. Transcript per million (TPM) counts provided by Kallisto for Alzheimer's disease (AD) and Progressive supranuclear palsy (PSP) samples for all *MAPT* transcripts identified. MT = MAPT p.A152T mutation carriers; WT = wild-type genotype.

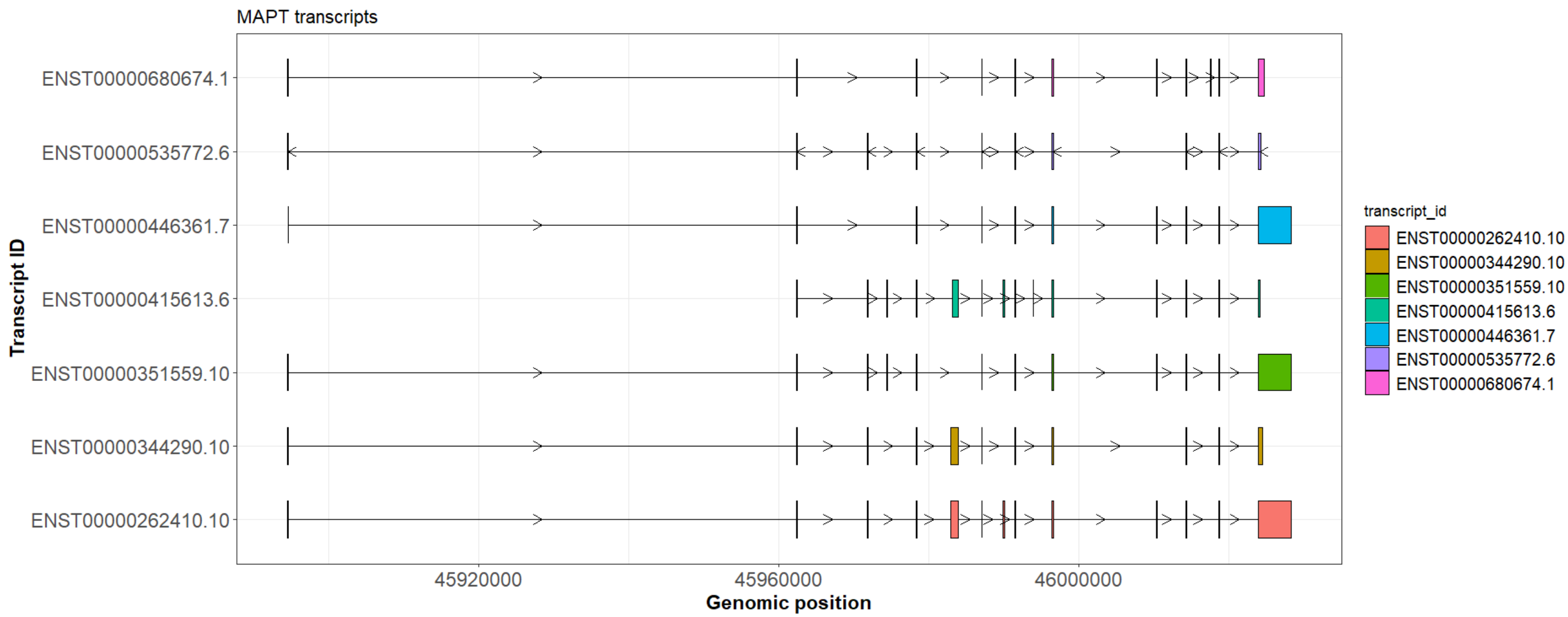

Supplementary Figure 1. Overview of protein-coding transcripts detected in bulk RNA sequencing using Kallisto and plotted using ggtranscript.

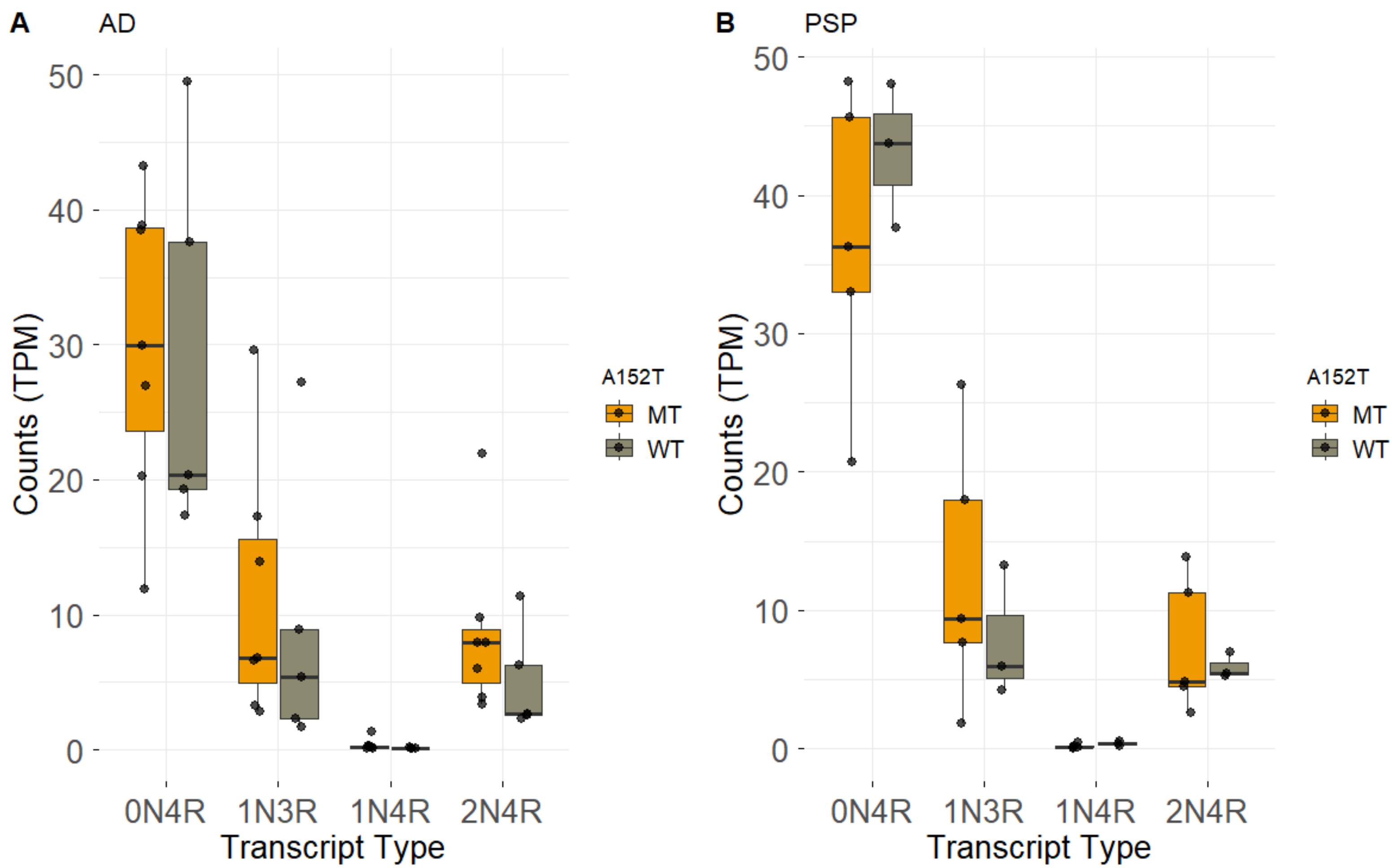

Supplementary Figure 2. Expression patterns of *MAPT* N-terminal variants in **(A)** Alzheimer's disease (AD; n=12) and **(B)** Progressive supranuclear palsy (PSP; n=8) samples colored by MAPT p.A152T variant status. Associations were assessed using linear regression models, both unadjusted and adjusted for sex and age at death. AD mutation carriers 2N4R median counts = 7.93. AD wildtype cases 2N4R median counts = 2.68. PSP mutation carriers 1N3R transcripts median counts = 9.35. PSP wildtype cases 1N3R median counts = 5.96. PSP mutation carriers 0N4R median counts = 36.3. PSP wildtype cases 0N4R median counts = 43.8. MT = MAPT p.A152T mutation carriers; WT = wild-type genotype. Individual points represent individual samples.
